# Predictive validation of a questionnaire for the assessment of social care needs in hospitalized older adults, the geriatric discharge complexity score: a diagnostic accuracy study

**DOI:** 10.1007/s40520-025-03194-2

**Published:** 2025-12-03

**Authors:** Andrea P. Rossi, Angela Scattolin, Leonardo Melchiori, Anna Goinavi, Andrea Pasqual, Katia Rossi, Serena Commissati, Matteo Bernardi, Valentina Muollo, Chiara Ceolin, Marina De Rui, Giuseppe Sergi

**Affiliations:** 1 Department of Medicine, Section of Geriatrics, AULSS 2, Treviso, Italy; 2 Department of Medicine, Cure Primarie, AULSS 2, Treviso, Italy; 3 Department of Neuroscience, Biomedicine and Movement, University of Verona, Verona, Italy; 4 Department of Medicine, Section of Geriatrics, University of Padua, Padua, Italy; 5 Division of Geriatrics, Healthy Aging Center Treviso, Department of Internal Medicine, Ospedale Cà Foncello, TREVISO, 31100 Italy; 6 Department of Medicine, University of Padua, Padua, Italy

**Keywords:** GDCS, BRASS index, Continuity of patient care, Discharge planning, Elderly care, Hospital stay, Nursing, Diagnostic accuracy study

## Abstract

**Background:**

Anticipating discharge challenges in the elderly population is essential to support effective care planning and reduce risks during care transitions. However, there is a lack of targeted assessment tools specifically designed for use in hospital wards to address this need.

**Aims:**

To evaluate the predictive value of the Geriatric Discharge Complexity Score (GDCS) and Blaylock Risk Assessment Screening Score (BRASS) with respect to discharge difficulties in a population of hospitalized older adults.

**Methods:**

The study was conducted on a sample of 416 subjects (175 females) with mean age of 88.2 ± 5.7 years. All subjects underwent evaluation with GDCS, Barthel scale, BRASS and count of total days of hospitalization and days of hospitalization related to social-welfare problems.

**Results:**

The GDCS showed sensitivity of 97.9% and specificity of 69.8% with an area under the curve (AUC) of 0.936. The BRASS showed sensitivity of 86.3% and specificity of 20.2% with an AUC of 0.518. Total and excess days of hospitalization for social problems were significantly higher in subjects with elevated GDCS.

**Conclusion:**

The GDSC seems more predictive of social care issues that may lead to a prolongation of hospital stay than the BRASS.

## Introduction

The aging population is projected to increase the demand for hospital capacity. Without improving the coordination of services following hospital discharge, this scenario will probably determine an increasing need for geriatric hospital beds and higher patient turnover rates. In this context, discharge planning, including medical and social needs, is pivotal [1]. 

According to findings by Selker et al., nearly one-third of discharges from internal medicine wards are subject to delays stemming from non-clinical causes.

This is primarily due to the lack of adequate discharge planning, poor knowledge of the patients’ social circumstances, social frailty identification and unavailability of post-discharge facilities [2–4]. 

Social frailty is defined as a state of increased vulnerability caused by limited social resources, weak support networks, or socioeconomic difficulties, which reduce an individual’s ability to manage health-related challenges [5]. 

Therefore, it is crucial to identify all factors that could make patient’s early discharge problematic or complex, so that discharge planning and specific actions can be undertaken in a timely manner.

The Blaylock Risk Assessment Screening Score (BRASS) is used broadly in clinical practice to identify patients at risk of prolonged hospitalization and reduce post-discharge problems, but only a few items are related to the patient’s social context [6, 7]. 

For this specific purpose, the Geriatric Discharge Complexity Score (GDCS), an 8-item questionnaire, was developed to assess the weaknesses of the healthcare and socio-economic support network of older adults admitted to acute wards.

**Fig. 1:**
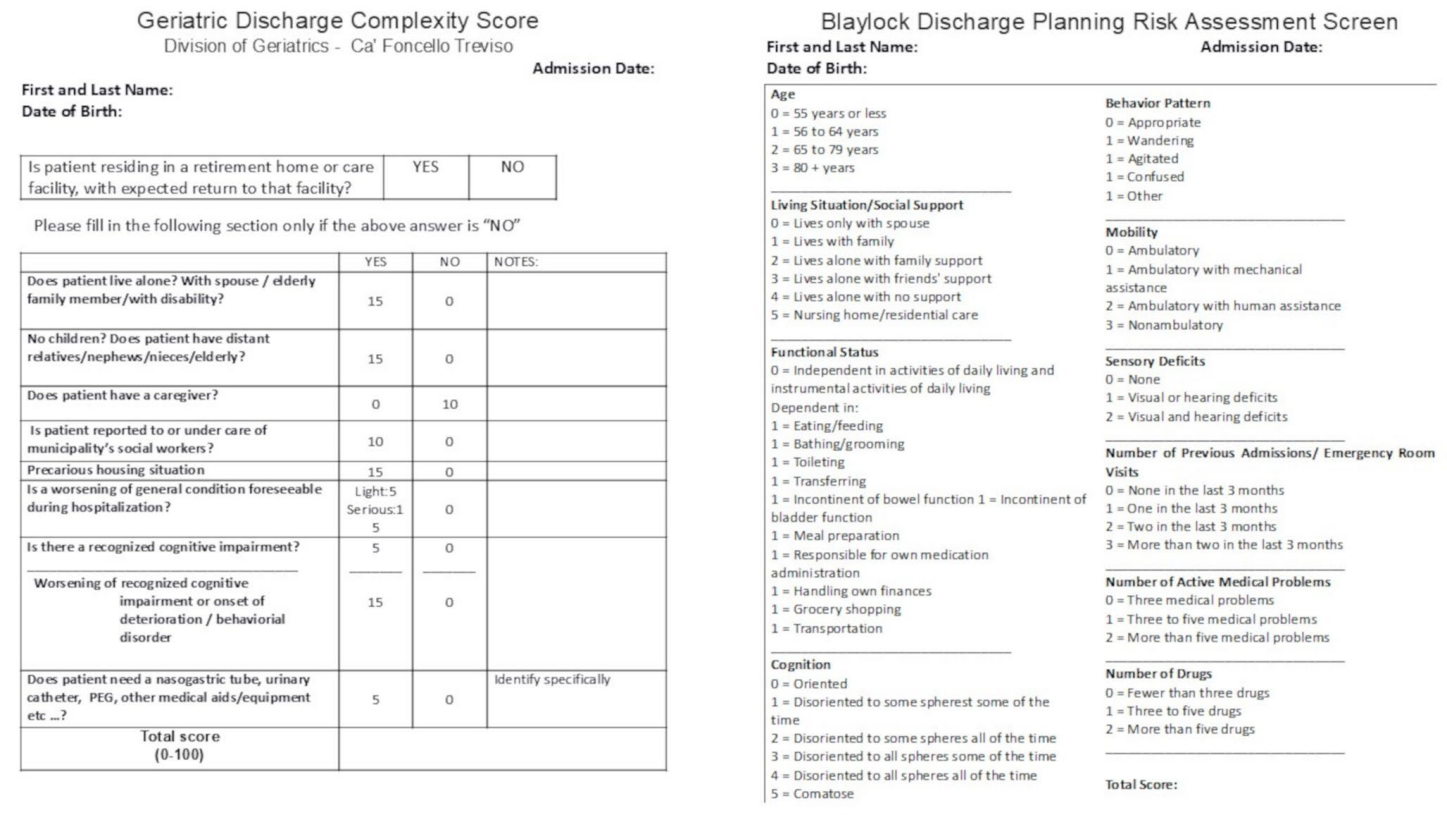
Shows the GDCS questionnaire that was submitted to the family members of the hospitalized patients and the BRASS

A side-by-side comparison of the GDCS and BRASS items, together with available validation outcomes, is reported in Fig. 1.

The GDCS was administered to family members and patients during the first days of hospitalization in the Geriatric ward of Treviso, in order to identify patients with social-welfare issues and possible discharge complications in order to avoid the prolongation of hospital stay for reasons not related to patients’ health conditions.

Therefore, the primary aim of this study is to assess the predictive value of the GDCS regarding actual discharge difficulties in geriatric patients admitted to an acute ward, in comparison with the BRASS, widely used screening tools for social frailty and post-discharge problems [6, 7]. A secondary aim is to evaluate length of stay (LOS) and in-hospital mortality in this population. In fact, as previously shown by Zarovska et al., higher BRASS scores indicate a greater risk of hospital mortality, too if the score’s predictive ability is lower for hospital mortality than for complex discharge [8]. We therefore decided to test our questionnaire also with respect to this important outcome.

## Methods

### Study population

The study was conducted on a sample of 416 subjects (175 females and 241 males) with a mean age of 88.2 ± 5.7 years, consecutively recruited from the Geriatric Ward of the Department of Medicine at the Treviso Hospital between April and December 2022. Subjects living in nursing homes (*n* = 49) were excluded from the present analysis.

Information on patients’ demographic profile, social context (such as age, household composition, and available support network), and clinical status — including cognitive abilities, functional mobility, sensory limitations, behavioral traits, prior hospitalizations, ongoing medical issues, and current medications — was obtained through structured interviews with the patient or, when necessary, their caregiver.

### Geriatric discharge complexity score

The Geriatric Discharge Complexity Score (GDCS) is composed of 8 items regarding (1) familiar assessment (three questions related to marital situation, presence of children or grandchildren, presence of another caregiver), (2) social assessment (two questions related to previous referrals to social services, living conditions), (3) clinical assessment (three questions related to worsening during hospitalization, presence of cognitive impairment and presence of nasogastric tube, urinary catheter or percutaneous endoscopic gastrostomy, PEG) selected on the basis of a review of the literature regarding risk factors for adverse hospitalization outcomes in older adults, including both clinical predictors and domains of social frailty such as family support, living arrangements, and prior involvement of social services [1–4, 6, 7]. An arbitrary score has been assigned to each item (Table 1).

**Table 1:** Association of specific GDC score items with discharge outcome

	Number of subjects (%)		Weight	Complex discharge			
				OR	95% CI	*P*	Regression coefficient (β)
**Does patient live alone? With spouse / elderly family member/with disability?**	181 (43.5%)	No Yes	0 15	1 17.63	9.00-34.53	< 0.001	2.87
**No children? Does patient have distant relatives/nephews/** **nieces/elderly?**	63 (15.1%)	No Yes	0 15	1 11.32	6.21–20.62	< 0.001	2.43
**Does patient have a caregiver not from his/her family?**	304 (73.1%)	No Yes	10 0	2.81 1	1.50–5.29	= 0.001	1.01
**Is patient reported to or under care of municipality’s social workers?**	51 (12.2%)	No Yes	0 10	1 2.49	1.35–4.62	= 0.004	0.91
**Precarious housing situation**	38 (9.1%)	No Yes	0 15	1 7.41	1 3.65–15.04	< 0.001	2.00
**Is a worsening of general condition foreseeable during hospitalization?**	181 (43.5%) 132 (31.7%)	No Yes, Mild Yes, Severe	0 5 15	1 2.33 7.01	1.07–5.09 3.25–15.09	< 0.05 < 0.001	0.84 1.95
**Is there a recognized cognitive impairment?**	173 (41.6%)	No Yes	0 5	1 1.13	0.64–2.02	0.667	0.13
**Worsening of recognized cognitive impairment or onset of deterioration / behaviorial disorder**	88 (21.1%)	No Yes	0 15	1 4.14	2.27–7.54	< 0.001	1.42
**Does patient need a nasogastric tube**,** urinary catheter**,** PEG**,** other medical aids/equipment etc. …?**	278 (66.8%)	No Yes	0 15	1 1.10	0.67–1.79	0.707	0.94

GDC score: Geriatric Discharge Complexity score, OR: Odds ratio, PEG: percutaneous endoscopic gastrostomy

**Table 2:** Diagnostic performance of GDCS (8-item and 6-item versions) and BRASS for predicting discharge difficulties (*n* = 416; cases = 95; non-cases = 321)

Screening Tool (Cut-off)	TP	FN	FP	TN	Sensitivity % (95% CI)	Specificity % (95% CI)	PPV % (95% CI)	NPV % (95% CI)
**GDCS – 8 items (≥ 30)**	93	2	97	224	97.9 (92.6–99.4)	69.8 (64.5–74.5)	48.9 (41.9–56.0)	99.1 (96.8–99.8)
**GDCS – 6 items (≥ 25)**	86	9	75	246	90.5 (83.0–94.9)	76.6 (71.7–80.9)	53.4 (45.7–61.0)	96.5 (93.4–98.1)
**BRASS (> 20)**	82	13	256	65	86.3 (78.0–91.8)	20.2 (16.2–25.0)	24.3 (20.0–29.1)	83.3 (73.5–90.0)

GDC score: Geriatric Discharge Complexity score, BRASS: TP = true positive; FN = false negative; FP = false positive; TN = true negative; PPV = positive predictive value; NPV = negative predictive value. CIs are Wilson 95%

The GDCS was administered to family members and patients during the first days of hospitalization in the Geriatric ward of Treviso.

### BRASS and Barthel index

All subjects were screened within 48 h from admission with the BRASS, as well as the GDCS [6, 7]. The BRASS includes 10 domains: age, living situation, social support, functional status, cognitive status, comorbidities, behavior, mobility, number of previous hospitalizations, and medication use. Each domain contributes to a cumulative score that stratifies patients into low, moderate, or high risk of discharge difficulties [7].

The Barthel Index, a 10-item scale, was also administered at the beginning and at the end of hospitalization [9]. It evaluates independence in basic activities of daily living, including feeding, bathing, grooming, dressing, bowel and bladder control, toilet use, transfers, mobility, and stair climbing. Scores range from 0 (complete dependence) to 100 (full independence), with higher scores reflecting greater functional autonomy.

All tests were performed by a single trained operator, a medical doctor (AS), blinded to other results.

### Length of stay and discharge difficulties evaluation

At the end of the hospitalization, the count of total days of hospitalization and the days of hospitalization related to discharge difficulties after medical problems had been resolved were reported by the medical doctor in charge of the patient. Patient discharge records were used to collect details on discharge method, destination, and length of hospitalization.

Similarly to Zarovska, we classified the discharge modality (primary study outcome) into three categories [8]:

- Discharged home without complications, including all patients discharged home with a LOS lower than the 90th percentile of diagnosis related groups-specific LOS observed in Veneto region hospitals;
- Complex discharge, including all alive patients not discharged home or patients discharged home with a LOS greater than the 90th percentile of diagnosis related groups specific LOS observed in Veneto region hospitals in the same year;
- Died in hospital.

### Statistical analysis

As a first step, we assessed the predictive performance of GDCS and BRASS scores treated as continuous variables.

Their capacity to discriminate between different discharge outcomes was examined through the area under the receiver operating characteristic (ROC) curve, with the AUC (area under the curve) computed for both scores.

To assess the statistical significance of the AUCs and compare ROC curves, we applied the method proposed by DeLong et al. [10] The “best” threshold for the GDCS for discharge difficulties was evaluated locating the point that maximizes the sum of sensitivity and specificity (i.e. the Youden index).

Subsequently, a revision of the original 8-items GDCS was conducted according to the different importance of each item (predictor).

To assess whether the predictive power of the excluded variables was accounted for by those retained, a binary logistic regression analysis was performed. The scores were based on regression coefficients and converted proportionally into integer numbers to the lowest coefficient satisfying two conditions: an odd ratio (OR) ≤ 1.20 and a *p*-value < 0.10. The two questions “Is there a recognized cognitive impairment?” and “Does patient need a nasogastric tube, urinary catheter, PEG, other medical aids/equipment etc …?” were therefore excluded from the simplified 6-items GDCS. The updated and simplified 6-item GDCS was calculated for each patient using the new scoring rule with the relative AUC. The final simplified model was used to derive two separate scores, the 8-item and the 6-item score, to predict better the two different outcomes: (a) complex discharge and (b) in-hospital mortality.

## Results

Mean LOS was 12.1 ± 7.3 days and the number of days of hospitalization not related to medical reasons was 1.29 ± 3.7 days. The association of specific items with discharge outcomes is shown in Table 1.

**Fig. 2:**
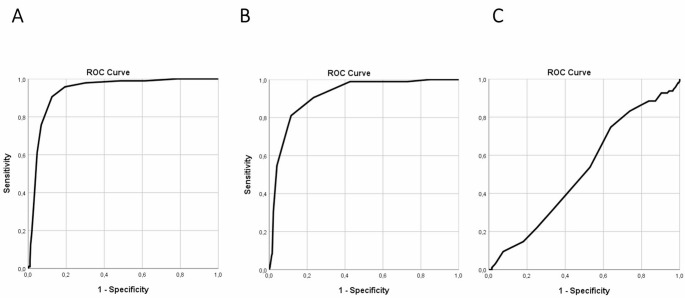
Shows the area under the curve related to discharge difficulties of 8-ites GDCS (**A**), 6-item GDCS (**B**) and BRASS (**C**)

The ability to predict the risk of discharge difficulties of the GDCS and BRASS questionnaires was evaluated by comparing their ROC curves. The AUC was 0.936, 0.915, and 0.518 for the 8-item GDCS, the 6-item GDCS and the BRASS, respectively (Fig. 2). The AUC of the short version of the GDCS was not significantly lower than that of the 8-item GDCS.

In the study population, which considered discharge difficulties as the outcome, the optimal cut-off that obtained better sensitivity and specificity values was calculated using the Youden index, and corresponds to scores ≥ 30 for the 8-item and ≥ 25 for the 6-item GDCS. Regarding those cutoffs, the sensitivity and specificity calculation of the 8-item GDCS questionnaire was 97.9% and 69.8%, respectively, while those for the 6-item questionnaire were 90.5% and 76.6%, respectively (Table 2). For the BRASS, a cutoff > 20 was used, which is recognized as indicative of high risk for discharge difficulties. In our study population, the BRASS showed a sensitivity of 86.3% and specificity of 20.2% (Table 2).

The DeLong test indicated a highly significant difference in AUC between the 8-item GDCS (AUC = 0.936) and the BRASS (AUC = 0.518), with a Z-score of 11.929 and a *p*-value < 0.001, which demonstrated the substantially better performance of the 8-item GDCS. Smilarly, the DeLong test revealed a statistically significant difference between the AUCs of the 6-item GDCS (AUC = 0.915) and the BRASS (AUC = 0.518), with a Z-score of 10.972 and a *p*-value < 0.001, which indicated that the 8-item GDCS demonstrated significantly better overall performance.

Conversely, no difference was observed comparing the AUC of the 6-item and 8-item GDCS (Z-score: 0.608, p: 0.543).

Subjects with GDCS ≥ 30 showed higher LOS as compared with individuals with a low GDCS: 14 days and 10 days, respectively. Correspondingly, a higher number of days of hospitalization related to discharge difficulties was observed in subjects with elevated GDCS as compared with those with a low score: 3 against 0 days, respectively. Conversely, no differences in the Barthel index and age were observed between subjects with high or low GDCS.

Conversely, no significant differences in both total days of hospitalization and the number of days related to discharge difficulties were observed by dividing the study population based on the BRASS.

AUCs related to in-hospital mortality were 0.441, 0.446 and 0.598 for the 8-item GDCS, the 6-item GDCS and the BRASS, respectively.

## Discussion

The results of our study suggest that the GDCS has better discriminative and predictive ability as compared with the BRASS. Moreover, in our study population, a GDCS ≥ 30 was associated with longer LOS and a higher number of days of hospitalization related to discharge difficulties as compared to the BRASS.

Previous studies showed that the BRASS is a useful, easy to use and reliable tool in identifying patients at increased risk of prolonged hospitalization, in planning more appropriate discharges, and in reducing or preventing post-discharge problems [7, 11, 12], but our results show that both versions of the GDCS seem to have better predictive power in identifying subjects at risk of discharge difficulties. In this regard, it is important to note that the BRASS is not specifically designed to evaluate older adults, whilst the GDCS score is. The BRASS index has good specificity in highlighting problems related to hospital discharge, but it has a low sensitivity. Brass includes assessment of comorbidities, number of medications and functional status that only indirectly affect the patient’s re-entry difficulties, which GDCS does not consider. Furthermore, BRASS considers the presence of previous institutionalization or home care living as a negative factor, thus assigning a high score, but those factors facilitate the discharge of the patient.

Conversely, the BRASS was more predictive of in-hospital mortality as compared with both versions of the GDCS and this is in line with the fact that BRASS also includes age, mobility, number of previous admission/emergency room visits, number of active medical problems and current drug regimes [13]. This is not surprising considering that the GDCS was conceived to detect socio-economic fragilities affecting discharge planning, and not to predict in-hospital mortality and focuses exclusively on discharge-related challenges.

The high sensitivity of the GDCS suggests that it can be effectively used as a screening tool to rule out patients not at risk of discharge difficulties. Although its specificity is lower, the implications of false positives are limited compared to the consequences of missing a truly at-risk patient. Therefore, a positive GDCS result should prompt further assessment and proactive discharge planning, while a negative result can reliably exclude the need for additional interventions.

Considering that the GDCS is based on a reduced number of questions as compared with the BRASS (6 or 8-items and 20-items, respectively), we can suppose that both versions of the GDCS take less time to be applied by healthcare workers. In fact, the GDCS short form has only 6 items and therefore can be administered even more quickly, with only slightly lower sensitivity and higher specificity, as compared with the 8-item version, considering that the 6-item GDCS showed an AUC that was not statistically different from the 8-item version. Therefore, a more parsimonious version of the GDCS with only 6-items seems to maintain the same capacity to identify subjects at risk for discharge difficulties. This finding, however, should not be interpreted as proof of equivalence, since the study was not powered to detect small differences between two closely related instruments and larger, independent cohorts will be required to determine whether the two versions perform equally well.

Aging of the Italian population and the rapid crumbling of the social fabric are urgent problems, characterized by the increase of small households, people living alone and low economical resources [14]. In our study population, those items related to social support, and in particular the absence of a caregiver, are the main risk factors for the prolongation of their hospitalization for non-medical reasons. This is not surprising and in line with risk factors for discharge difficulties previously described by Carey et al. [4]

The GDCS, more than the BRASS, underlines the socio-economic fragility that could be used in future research to identify earlier the best setting for each patient at the time of hospital discharge. In addition, all items of the GDCS could be used to develop and employ more appropriate and specific welfare pathways and services that could respond to people’s socio-economic needs. In this perspective, the early identification of patients with social care needs through the GDCS may also support proactive care planning. A timely involvement of primary care services and closer collaboration between hospital teams, community health services, and social workers could facilitate the organization of post-discharge support. Such integration may improve continuity of care, prevent unnecessary prolongation of hospital stay, and optimize the use of hospital resources.

This study has several limitations that should be acknowledged. Firstly, both the derivation of the optimal cut-off and the estimation of diagnostic performance were performed in the same population, which may have led to overly optimistic estimates of sensitivity and specificity. External validation in independent and multicenter cohorts is therefore required before the GDCS can be recommended for routine clinical use.

Secondly, this is the first study to propose and evaluate the GDCS in a clinical setting. Other psychometric properties, such as construct validity, inter-rater reliability, and reproducibility, have not yet been established and warrant further investigation. Thirdly, it was conducted in the context of Italian acute care facilities, where social support systems and post-discharge services are organized in a specific way. Since these arrangements vary considerably across regions, health care systems, and countries, the generalizability of our findings to other settings, especially at the international level, may be limited. Fourthly, this was a single-centre study, which, while providing valuable real-world data, may not be entirely reproducible in different hospitals or care environments. Lastly, the cut-offs for the GDCS were derived and tested within the same study sample. This methodological aspect implies that the diagnostic performance parameters (such as sensitivity and specificity) should be regarded as preliminary estimates, and confirmation in independent and larger multicentric cohorts is needed.

In conclusion, these preliminary data seem to indicate that the GDCS is more predictive of social care problems that can lead to prolongation of hospital stay than the BRASS. The early identification of patients at risk for not health-related discharge problems, from the moment of hospital admission, can help to implement appropriate discharge strategies. This approach can reduce hospitalization length, not directly related to clinical problems, and result in better resource allocation.

## Data Availability

No datasets were generated or analysed during the current study.
